# Association of Anesthetic Exposure Time With Clinical Outcomes After Endovascular Therapy for Acute Ischemic Stroke

**DOI:** 10.3389/fneur.2019.00679

**Published:** 2019-06-26

**Authors:** Lorenz Raming, Haidar Moustafa, Alexandra Prakapenia, Jessica Barlinn, Johannes Gerber, Hermann Theilen, Timo Siepmann, Lars-Peder Pallesen, Kevin Haedrich, Simon Winzer, Heinz Reichmann, Jennifer Linn, Volker Puetz, Kristian Barlinn

**Affiliations:** ^1^Department of Neurology, Carl Gustav Carus University Hospital, Technische Universität Dresden, Dresden, Germany; ^2^Institute of Neuroradiology, Carl Gustav Carus University Hospital, Technische Universität Dresden, Dresden, Germany; ^3^Department of Anesthesiology, Carl Gustav Carus University Hospital, Technische Universität Dresden, Dresden, Germany

**Keywords:** endovascular therapy, stroke, anesthetics, sedation, neurocritical care

## Abstract

**Background:** The optimal sedative regimen with general anesthesia (GA) or conscious sedation for patients undergoing endovascular therapy (EVT) remains controversial. Apart from sedative regimen, the duration of anesthetic exposure may affect clinical outcomes. We aimed to determine whether there is an association between anesthetic exposure time and clinical outcomes in mechanically ventilated stroke patients undergoing EVT for large vessel occlusion.

**Methods:** This was an observational study of consecutive ischemic stroke patients who underwent EVT for anterior circulation large vessel occlusion under GA from January 2016 to March 2018. To minimize confounding by indication, we restricted our analysis to patients whose anesthetic exposure lasted <72 h. Multivariable logistic regression modeling adjusted for covariates was employed to evaluate whether 90-days independent functional outcome (defined as modified Rankin Scale scores 0–2) and 90-days survival could be predicted by anesthetic exposure time.

**Results:** During the study period, 138 patients with ischemic stroke who underwent EVT received GA and fulfilled our study criteria: median age was 77 years (interquartile range, 65–82); 46.4% were men; median NIHSS score was 18 (15–21), median ASPECT score was 7 (6–8). Median duration of GA was 5.4 (2.5–19.7) h. Logistic regression modeling revealed an independent association between duration of anesthetic exposure and both 90-days independent functional outcome (*p* = 0.034) and 90-days survival (*p* = 0.011). Each additional 15-min of anesthetic exposure decreased the likelihood of achieving an independent functional outcome at 90 days by 1.5% and of being alive at 90 days by 1.0%.

**Conclusion:** Our data promotes the notion that ischemic stroke patients who require peri-interventional GA for EVT should be extubated as soon as possible after the procedure.

## Introduction

Safety and efficacy of endovascular therapy (EVT) for acute ischemic stroke caused by large vessel occlusion in the anterior circulation has been demonstrated by multiple randomized trials (1). However, most of the patients undergoing EVT require some kind of sedation or anesthesia to ensure calmness during the procedure, especially those suffering from severe stroke, and agitation (2). While earlier observational studies and a *post-hoc* analysis of the MR CLEAN trial pointed to detrimental effects of anesthesia on clinical outcomes in stroke patients undergoing EVT (3, 4), three single-center randomized trials recently demonstrated comparable clinical and imaging outcomes among stroke patients treated under general anesthesia (GA) or conscious sedation (CS) (5–7). Conversely, a recently published individual patient data meta-analysis of seven EVT randomized trials underpinned worse outcomes in stroke patients treated under GA compared with those treated under CS or local anesthesia (8). Thus, the optimal sedative regimen for patients undergoing EVT remains a matter of controversial debate (9).

In experimental research, anesthesia has been shown to worsen ischemic outcome indirectly by causing hypotension or impaired cerebral autoregulation (10). Nonetheless, from a bedside perspective such potential detrimental effects of anesthesia may follow a time- and dose-dependent relationship as ischemic penumbra may persist for many hours depending on collateral and hemodynamic status (11). Most patients in the single-center GA vs. CS trials were extubated immediately after the EVT procedure, limiting their anesthetic exposure to the time spent in the neuro-interventional suite (5–7). From the EVT randomized trials, however, it is not reported how many of the intubated patients required continuation of anesthesia after completion of EVT (8).

In real-world scenario, it can be challenging to extubate patients immediately after the EVT procedure as many patients require further sedation and airway protection due to severity of cerebral ischemia. In unselected critically ill patients treated on an intensive care unit, deep sedation has been accepted as prognostic variable for in-hospital and follow-up mortality (12, 13). In addition, duration of ventilation following EVT in stroke patients has been linked to ventilator-associated complications and unfavorable outcome (14). It remains to be answered if potential detrimental effects of anesthesia in stroke patients, if any, follow an all-or-nothing principle or a time-dependent relationship.

We therefore aimed to test the hypothesis that prolonged exposure to anesthetics is associated with unfavorable clinical outcomes in mechanically ventilated stroke patients undergoing EVT for anterior circulation large vessel occlusion.

## Methods

### Study Design and Population

This was an observational study based on our prospective registry of ischemic stroke patients ≥18 years who underwent EVT for anterior circulation large vessel occlusion between January 2016 and March 2018. According to our guideline-guided institutional protocol, acute ischemic stroke patients directly admitted or transferred from an outside hospital to our tertiary-care stroke center were considered EVT-eligible, if the following criteria were met: elapsed time from symptom onset ≤ 6 h (adopted to ≤ 24 h after publication of DAWN and DEFUSE-3 data), baseline non-contrast computed tomography ASPECTS ≥6 points, CT-angiography (CTA) demonstrating a symptomatic intracranial occlusion of the middle cerebral (MCA) and/or internal carotid artery (ICA), and in selected cases, evidence of potentially beneficial CT-perfusion maps, and/or beneficial collateral circulation on CTA (15–17). Patients who were transferred from an outside hospital routinely underwent repeat imaging at our center to exclude extensive infarction/hemorrhage and confirm persistent large vessel occlusion. The final decision to perform EVT was at the discretion of the treating stroke neurologist and neuroradiologist. Patients who underwent EVT for large vessel occlusion in the posterior circulation were excluded from further evaluation in this study.

The decision whether to perform EVT under GA or CS was made by the treating neuroradiologist and anesthetist. Major reasons for induction of or conversion to GA were respiratory failure, loss of tracheal reflex, aspiration, and lack of patient cooperation. To minimize risk of aspiration GA was routinely initiated as rapid sequence induction. Choice of analgo-sedative and dosage was individually made by the anesthetist, whereby in most patients propofol, sevoflurane, midazolam, and remifentanil, either alone or in combination, were used for induction and maintenance of GA and CS. Blood pressure management during and after EVT procedure followed current stroke guidelines on blood pressure management in acute stroke and our institutional protocol with thresholds for systolic blood pressure set at 120–160 mmHg once recanalization was achieved (15). Reason for use of anesthetics over the end of EVT procedure (i.e., sedation needed post-procedure) was facilitation of mechanical ventilation in patients who were not yet ready to extubate per judgment of the treating anesthetist or neuro-intensivist (e.g., presumed risk of aspiration, bridging time until follow-up CT to exclude large hemispheric infarction, agitation/asynchronous ventilation but simultaneous need of airway protection, pulmonary complications), or for safety reasons avoiding overnight extubation based on the judgment of the treating neuro-intensivist. To minimize confounding by indication bias we excluded patients whose exposure to anesthetics lasted ≥72 h (e.g., due to manifest large hemispheric infarction and management of increased intracranial pressure, severe clinical conditions necessitating prolonged airway protection) from this analysis.

Demographics (age, sex), vascular risk factors (hypertension, diabetes mellitus, dyslipidemia, atrial fibrillation, current smoking), admission National Institutes of Health Stroke Scale (NIHSS) and data related to brain imaging, acute treatment and time metrics (onset-to-needle, door-to-needle, door-to-imaging, door-to-groin puncture, onset-to-groin puncture, picture-to-groin puncture) were prospectively recorded for all patients. Groin-to-reperfusion procedural time was prospectively recorded from January 2017 onwards. Admission ASPECT scores on non-contrast CT, baseline status of the cerebral and pre-cerebral vasculature and reperfusion status post-EVT [classified by modified Thrombolysis in Cerebral Infarction [mTICI] score] were prospectively assessed by neuroradiologists. Non-contrast CT was performed in all patients 24 h post ictus as standard of care and additionally in case of clinically relevant neurological worsening. Duration of anesthesia and endotracheal intubation was calculated from documented times (start and end of anesthesia exposure, intubation and extubation) in the periprocedural anesthesia protocols and intensive care unit patient charts. Peri-interventional arterial hypotension was defined as systolic blood pressure <90 mmHg at any time during intervention as documented per anesthesia protocol. Peri-interventional desaturation was defined as oxygen saturation <90% as documented per anesthesia protocol.

Endpoints of interest were complete reperfusion post-EVT (defined as mTICI score of 2b or 3), modified Rankin Scale (mRS) scores obtained by telephone interview at 90 days, here independent functional outcome was defined as mRS scores ≤ 2, as well as in-hospital and 90-days survival. Safety endpoints comprised symptomatic intracerebral hemorrhage (sICH) post-treatment [defined according to ECASS-II criteria as imaging evidence of ICH with an increase ≥4 points in the NIHSS score (18)], in-hospital pneumonia (based on radiologic, laboratory and clinical findings, and eventually judged by the treating physician) and periprocedural complications such as vessel injury and secondary cerebral ischemia.

The study complied with The Strengthening the Reporting of Observational Studies in Epidemiology (STROBE) statement (19) and has been approved by the institutional research ethics committee of the Technische Universität Dresden (#272072017). Since we used observational data from an ongoing registry informed formal consent was not required; however, patients or their legally authorized representatives gave approval for treatment with intravenous thrombolysis and/or EVT, where possible.

### Statistical Analysis

Continuous and non-continuous variables are presented as mean (± standard deviation), median (interquartile range, IQR) and percentage. To describe our study population and potential confounding, we first compared demographics, clinical and outcome variables including 90-days independent functional outcome, 90-days survival and symptomatic ICH between GA and CS patients. Corresponding between-group comparisons were conducted with the use of chi-square test, Fisher's exact test and Mann-Whitney *U*-test, where applicable. Multivariable analysis was subsequently performed with the use of logistic regression to adjust for variable imbalances and known outcome predictors such as age, admission NIHSS, baseline ASPECTS, intravenous thrombolysis, and reperfusion post-EVT. In a further attempt to explore confounding by indication bias in GA patients, we categorized anesthesia exposure in time intervals of <3, 3–6, 6–12, 12–24, 24–48, and 48–72 h, and compared patients' baseline characteristics by these time intervals.

In a second step, we performed multivariable logistic regression analysis to explore the predictive value of anesthesia duration for above mentioned outcomes of interest. For this purpose, anesthetic exposure duration was categorized in 15- and 60- min strata, as these time intervals were considered clinically relevant. A priori selected variables known from literature as potential outcome predictors were chosen as covariates for our multivariable logistic regression model. The final model, however, was conducted using a backward selection procedure, whereas covariates not meeting significance level of *p* < 0.1 were removed from the model. To test robustness of the model, we repeated our multivariable regression analysis using a forward selection procedure. Complete-case analysis was applied for missing data imputation. Odds ratios (OR) are presented with corresponding 95% confidence intervals (CI). Significance level was set at *p* < 0.05. The statistical software package STATA (Version 12.1, StataCorp., College Station, TX) was used for statistical analysis.

## Results

### Study Population

**Figure 1:**
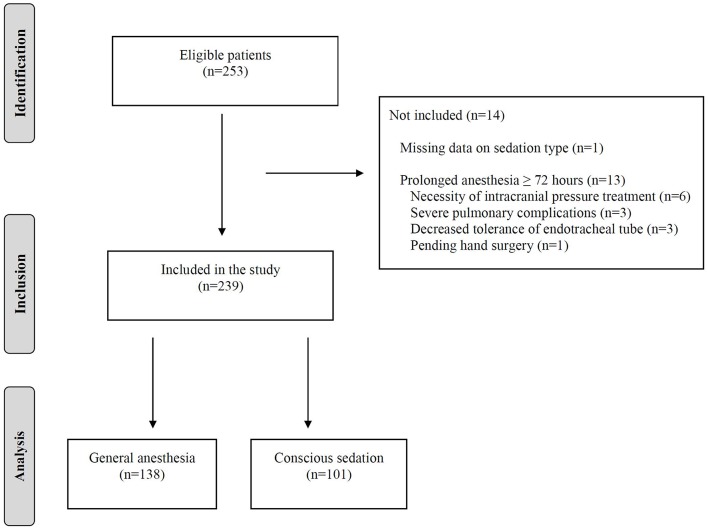
Study flow diagram.

During the study period of 27 months, 253 consecutive ischemic stroke patients underwent EVT for anterior circulation large vessel occlusion at our stroke center. Of these, 152 patients (60.1%) received GA initiated prior to or during the EVT procedure and 101 patients (39.9%) received CS. After exclusions, 138 patients who were treated under GA remained for analysis of anesthesia exposure duration and clinical outcomes. The flow diagram is depicted in Figure 1.

The majority of GA patients was intubated immediately prior to the EVT procedure, whereas five (3.7%) patients were anesthetized and intubated in the preclinical emergency setting. Twenty patients required conversion from CS to GA with agitation (40%) being the most frequent reason, followed by respiratory failure (30%), aspiration (10%), and miscellaneous causes (20%). The most frequently used anesthetics during EVT were propofol (91.9%), sevoflurane (5.8%), and midazolam (2.3%), while continuation of sedation after EVT was mostly accomplished with propofol (84.3%) followed by midazolam (2.4%) and a combination of both (13.3%). Significant peri-interventional systolic blood pressure drops occurred in four (2.9%) and oxygen desaturations in three (2.2%) patients. Median anesthetic exposure duration and endotracheal intubation was 5.4 (2.5–19.7) and 8.3 (3.2–32.3) h, respectively. Median time elapsed between the end of EVT-procedure and end of anesthetic exposure was 3.6 (0.8–18.4) h. Anesthetics were used for 0–3 h in 44/138 (31.9%) patients, 3–6 h in 26 (18.8%), 6–12 h in 19 (13.8%), 12–24 h in 22 (15.9%), 24–48 h in 23 (16.7%), and 48–72 h in 4 (2.9%) patients. Clinical and imaging characteristics by anesthesia exposure time intervals are detailed in Table 1. Reasons for sedation needed post-procedure were mostly related to safety measures (e.g., avoidance of overnight extubation) or patient incompliance/asynchronous ventilation with simultaneous need of airway protection due to presumed risk of aspiration. Two patients (1.4%) suffered from clinically asymptomatic non-ST-segment elevation myocardial infarction, which did not affect further clinical course. In 20 patients (14.5%) who developed pneumonia during hospital course, sedation was discontinued within 24 h in 11 and within 48 h in 19 patients. Only one patient with pneumonia required prolonged sedation for 49.2 h.

**Table 1:** Clinical and imaging characteristics by general anesthesia time intervals.

**Variable**	** <3 h (*n* = 44)**	**3–6 h (*n* = 26)**	**6–12 h (*n* = 19)**	**12–24 h (*n* = 22)**	**24–48 h (*n* = 23)**	**48–72 h (*n* = 4)**
Age, years, median (IQR)	74 (64–82)	79 (72–85)	79 (73–82)	75 (60–79)	79 (71–82)	71 (67–77)
Sex, men (%)	21 (47.7)	10 (38.5)	7 (36.8)	11 (50)	14 (60.9)	1 (25)
Admission NIHSS–score, median (IQR)	17 (14–19)	19 (15–21)	17 (12–21)	20 (17–21)	19 (15–21)	15 (11–17)
Intravenous thrombolysis, *n* (%)	20 (45.5)	13 (50)	12 (63.2)	14 (63.6)	16 (69.6)	1 (25)
**VASCULAR RISK FACTORS**, ***n*** **(%)**						
Hypertension	34 (77.3)	24 (92.3)	19 (100)	18 (81.8)	22 (95.7)	3 (75)
Diabetes mellitus	14 (31.8)	10 (38.5)	7 (36.8)	9 (40.9)	8 (34.8)	2 (50)
Dyslipidemia	12 (27.3)	6 (23.1)	10 (52.6)	7 (31.8)	4 (17.4)	0
Atrial fibrillation	20 (45.5)	19 (73.1)	16 (84.2)	10 (45.5)	18 (78.3)	2 (50)
Current smoking	9 (20.5)	2 (7.7)	0	1 (4.6)	1 (4.4)	0
Previous cerebral ischemia	2 (4.6)	4 (15.4)	4 (21.1)	4 (18.2)	2 (8.7)	0
Baseline serum glucose, mmol/L, median (IQR)	7.7(6.4–9.1)	8.2 (6.9–9.1)	7.3 (6.6–9.8)	6.7 (5.6–9.1)	7.1 (6.4–9.6)	9.3 (7.9–10.1)
**IMAGING**						
Admission ASPECTS, median (IQR)	7 (6–8)	7 (7–8)	7 (6–9)	7 (6–7)	7 (6–8)	8 (7–8)
Occlusion site, *n* (%)						
ICA-MCA	11 (25)	4 (15.4)	6 (31.6)	6 (27.3)	7 (30.4)	1 (25)
M1-MCA	30 (68.2)	22 (84.6)	13 (68.4)	14 (63.6)	14 (60.9)	3 (75)
M2-MCA	3 (6.8)	0	0	2 (9.1)	2 (8.7)	0
Reperfusion post-EVT (TICI 2b/3), *n* (%)	35 (79.6)	22 (84.6)	18 (94.7)	17 (77.3)	16 (69.6)	3 (75)
**TIMES METRICS, MIN, MEDIAN (IQR)**						
Onset-to-needle	95 (71–110)^†^	127 (106–163)^†^	128 (102–195)^††^	110 (103–141)	124 (85–139)	72 (–)
Door-to-needle	45 (34–61)	49 (37–75)^†^	50 (42–78)^§^	49 (38–65)	41 (35–54)^††^	24 (–)
Door-to-imaging pre-EVT	15 (9–3)^‡^	15 (8–23)	12 (7–16)	19 (15–25)	16 (13–22)	12 (10–14)
Onset-to-groin puncture	238 (190–295)^||^	266 (190–310)^||^	350 (270–370)^#^	265 (173–325)^*^	272 (214–330)	276 (222–336)
Door-to-groin puncture	78 (58–111)	68 (51–100)	74 (60–90)	77 (67–86)	91 (71–117)	68 (51–94)
Picture–to–groin puncture	59 (47–81)	56 (43–88)	59 (50–77)	54 (44–74)	76 (62–98)	54 (41–80)
Groin-to-reperfusion	55 (40–76)^§§^	56 (48–93)^‡‡^	52 (47–90)^##^	62 (56–84)^**^	58 (50–78)^Δ^	121 (113–174)^$^

*IQR, indicates interquartile range; NIHSS, National Institutes of Health Stroke Scale; ASPECTS, Alberta Stroke Program Early CT Score; ICA, internal carotid artery; MCA, middle cerebral artery; TICI, Thrombolysis in Cerebral Ischemia*.

† missing data for 1 patients;

†† missing data for 2 patients;

§ missing data for 3 patients;

‡ missing data for 1 patient (non-assessable due to in-hospital stroke);

|| missing data for 4 patients (non-assessable due to unknown stroke onset);

# missing data for 2 patients (non-assessable due to unknown stroke onset);

* missing data for 1 patient (non-assessable due to unknown stroke onset);

§§ missing data for 18 patients;

‡‡ missing data for 13 patients;

## missing data for 7 patients;

** missing data for 8 patients;

Δ missing data for 16 patients;

$ *missing data for 1 patient*.

### Comparison of General Anesthesia and Conscious Sedation

Patients who received GA had more severe strokes (median NIHSS scores: 18 vs. 14 points; *p* = 0.0001) and more frequently diabetes mellitus (36.2% vs. 20.8%; *p* = 0.01) compared with patients in the CS group. Baseline clinical and imaging variables with no further differences in group-to-group comparisons are shown in Table 2. Data on 90-days functional outcome were available for 137 patients (99.3%) in the GA group and 100 (99%) in the CS group.

**Table 2:** Clinical and imaging characteristics for general anesthesia and conscious sedation groups.

**Variable**	**General anesthesia (*n* = 138)**	**Conscious sedation (*n* = 101)**	***p***
Age, years, median (IQR)	77 (65–82)	75 (66–81)	0.62
Sex, men (%)	64 (46.4)	44 (43.6)	0.67
Admission NIHSS-score, median (IQR)	18 (15–21)	14 (11–18)	0.0001
Intravenous thrombolysis	76 (55.1)	60 (59.4)	0.50
**VASCULAR RISK FACTORS**, ***n*** **(%)**			
Hypertension	120 (86.9)	89 (88.1)	0.79
Diabetes mellitus	50 (36.2)	21 (20.8)	0.01
Dyslipidemia	39 (28.3)	36 (35.6)	0.22
Atrial fibrillation	85 (61.6)	57 (56.4)	0.42
Current smoking	13 (9.4)	12 (11.9)	0.54
Previous cerebral ischemia	16 (11.6)	17 (16.8)	0.25
Baseline serum glucose, mmol/L, median (IQR)	7.5 (6.3–9.3)	7.2 (6.2–8.8)	0.18
**IMAGING**			
Admission ASPECTS, median (IQR)	7 (6–8)	7 (6–8)	0.54
Occlusion site, *n* (%)			0.67
ICA-MCA	35 (25.4)	17 (16.8)	
M1-MCA	96 (69.6)	76 (75.3)	
M2-MCA	7 (5.1)	8 (7.9)	
**TIMES METRICS, MIN, MEDIAN (IQR)**			
Onset-to-needle	110 (95–142)^†^	105 (82–136)^††^	0.29
Door-to-needle	46 (35–59)^§^	42 (32–58)^§^	0.33
Door-to-imaging pre-EVT	15 (10–23)^‡^	18 (12–26)^||^	0.09
Onset-to-groin puncture	262 (198–325)^#^	253 (183–328)^*^	0.47
Door-to-groin puncture	80 (60–104)^‡^	73 (55–88)^||^	0.08
Picture-to-groin puncture	62 (46–79)	53 (40–73)	0.007

*IQR, Indicates Interquartile Range; NIHSS, National Institutes of Health Stroke Scale; ASPECTS, Alberta Stroke Program Early CT Score; ICA, internal carotid artery; MCA, middle cerebral artery*.

† missing data for 4 patients;

†† missing data for 1 patient;

§ missing data for 6 patients;

‡ based on 137 patients (non-assessable due to in-hospital stroke, n = 1);

|| based on 95 patients (non-assessable due to in-hospital stroke or secondary large vessel occlusion, n = 6);

# based on 127 patients (non-assessable due to unknown stroke onset, n = 11);

* *based on 96 patients (non-assessable due to unknown stroke onset, n = 5)*.

Major reperfusion was similarly achieved in GA and CS groups (111/138 [80.4%] vs. 80/101 [79.2%]; *p* = 0.82). Independent functional outcome at 90 days was less frequently achieved in GA than in CS group (31/137 [22.6%] vs. 39/100 [39.0%]; *p* = 0.006). At discharge, GA patients were less frequently alive than non-GA patients (105/138 [76.1%] vs. 90/101 [89.1%]; *p* = 0.01). There was a trend toward 90-days stroke survival favoring the CS group (93/137 [67.9%] vs. 79/100 [79.0%]); however, this observation did not reach statistical significance (*p* = 0.058). No between-group differences were present with respect to periprocedural vessel injury (3/138 [2.2%] vs. 5/101 [4.9%]; *p* = 0.288), post-treatment symptomatic ICH (5/138 [3.6%] vs. 4/101 [4%]; *p* = 1.0), and in-hospital pneumonia (20/138 [14.5%] vs. 11/101 [10.9%]; *p* = 0.413).

In multivariable analysis adjusting for age, admission NIHSS, intravenous thrombolysis, admission ASPECTS, and reperfusion post-EVT, GA emerged as independent predictor of 90-days independent functional outcome (OR 1.99, 95%CI 1.02–3.87; *p* = 0.042), but not 90-days stroke survival (OR 1.51, 95%CI 0.78–2.93; *p* = 0.223).

When we restricted our analysis to the 44 patients whose anesthetic exposure lasted only 0 to 3 h (i.e., restricting analysis to EVT procedure), no differences in comparison to CS patients were present regarding 90-days independent functional outcome (18/44 [40.9%] vs. 39/100 [39.0%]; *p* = 0.829), in-hospital (37/44 [84.1%] vs. 90/101 [89.1%]; *p* = 0.40) and 90-days survival (34/44 [77.3%] vs. 79/100 [79.0%]; *p* = 0.816).

### Anesthetic Exposure Duration With Respect to Clinical Outcomes

**Figure 2:**
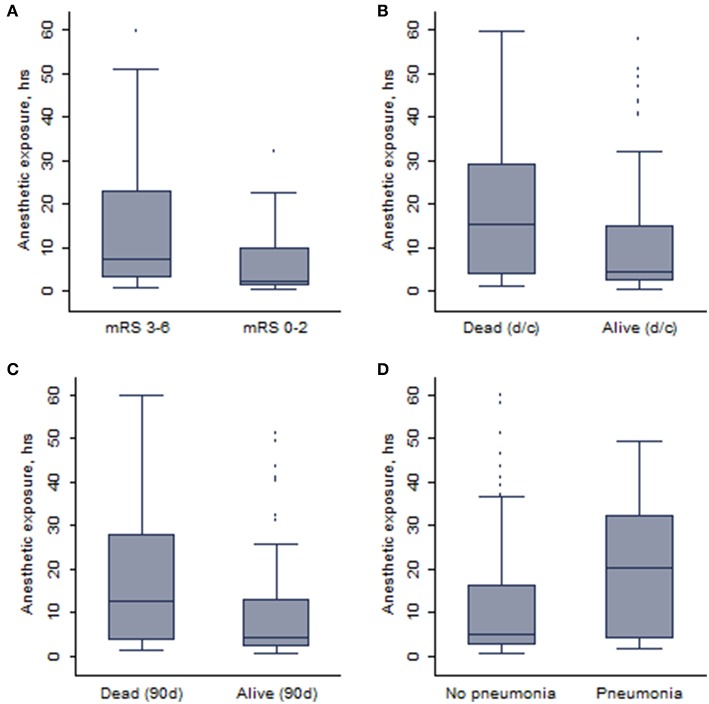
The whisker plots showing the association between duration of anesthetic exposure and **(A)** 90-days independent functional outcome, **(B)** survival at discharge (d/c), **(C)** 90-days survival, and **(D)** pneumonia.

In total, anesthetic exposure was shorter in patients who achieved an independent functional outcome (median: 2.4 vs. 7.8 h; *p* = 0.0002) or were alive (median: 4.5 vs. 11.4 h; *p* = 0.01) at 90 days. Similarly, patients who developed pneumonia (20/138, 14.5%) during hospitalization were exposed to anesthetics longer than those who did not develop pneumonia (median: 20.2 vs. 5.1 h; *p* = 0.006). Corresponding box and whisker plots are depicted in Figure 2. No difference in anesthetic exposure time was present with respect to occurrence of symptomatic ICH (median: 4.2 vs. 5.6 h; *p* = 0.72).

**Figure 3:**
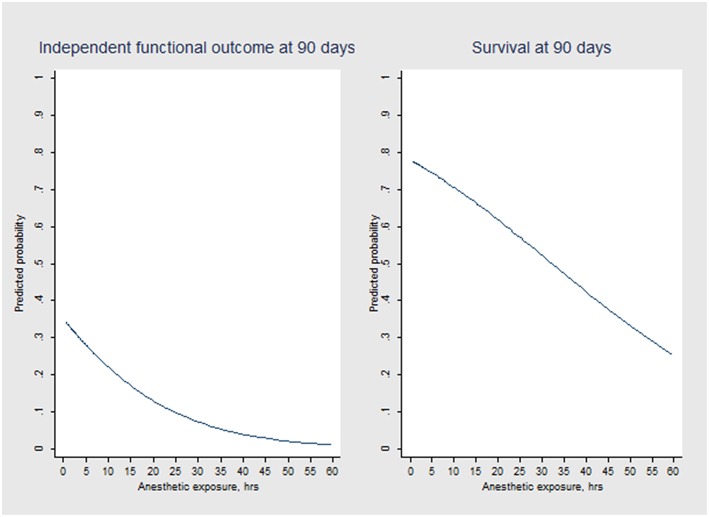
Predicted probability of independent functional outcome (mRS scores 0 to 2) and survival over anesthetic exposure duration according to logistic regression modeling.

Multivariable logistic regression analysis revealed an independent association between anesthetic exposure time and both 90-days independent functional outcome (*p* = 0.034) and 90-days survival (*p* = 0.011) (Tables 3, 4). In addition, each additional 15-min of anesthetic exposure decreased the likelihood of achieving an independent functional outcome at 90 days by 1.5%. Similarly, likelihood of being alive at 90 days decreased by 1.0% per each additional 15-min of anesthetic exposure. When we repeated our multivariable analysis considering only anesthetic exposure after the end of EVT-procedure (instead of using the entire anesthetic exposure time as factor variable), associations with 90-days independent functional outcome (adjusted OR: 0.95; CI95%, 0.89–0.99; *p* = 0.043) and 90-days survival (adjusted OR: 0.96; CI95%, 0.93–0.99; *p* = 0.009) remained unchanged. All these associations were reproduced after conducting a stepwise forward selection procedure for logistic regression analysis. Figure 3 illustrates predicted probability of favorable functional outcome and survival over duration of exposure to anesthetics.

**Table 3:** Associations between baseline and clinical variables and 90-days favorable functional outcome on stepwise backward Wald logistic regression analysis^*^.

**Variable**	**Comparison**	**OR (95% CI)**	***p***
Age	Per 1-year increase	0.93 (0.88–0.98)	0.007
Arterial hypertension	Yes vs. no	0.26 (0.07–0.99)	0.049
Admission-ASPECT score	Per 1-point increase	1.69 (1.14–2.51)	0.007
Diabetes mellitus	Yes vs. no	0.35 (0.11–1.16)	0.086
Occlusion site	M2 vs. M1 vs. MCA-ICA	2.84 (0.9–8.88)	0.074
Anesthetic exposure	Per 1-h increment	0.94 (0.89–0.99)	0.034

* *The following variables were included in the multivariable logistic regression model: age, arterial hypertension, diabetes mellitus, admission NIHSS-score, admission-ASPECT score, intravenous thrombolysis, occlusion site, onset-to-groin puncture time, reperfusion status post-EVT and duration of anesthetic exposure. Variables arriving at p>0.1 were removed from the model*.

**Table 4:** Associations between clinical variables and 90-days survival on stepwise backward Wald logistic regression analysis^*^.

**Variable**	**Comparison**	**OR (95% CI)**	***p***
Age	Per 1-year increase	0.93 (0.89–0.97)	0.002
Diabetes mellitus	Yes vs. no	0.36 (0.15–0.84)	0.019
Anesthetic exposure	Per 1-h increment	0.96 (0.93–0.99)	0.011

* *The following variables were included in the multivariable logistic regression model: age, arterial hypertension, diabetes mellitus, admission NIHSS-score, admission-ASPECT score, intravenous thrombolysis, occlusion site, onset-to-groin puncture time, reperfusion status post-EVT and duration of anesthetic exposure. Variables arriving at p > 0.1 were removed from the model*.

## Discussion

Our study showed an independent association between length of anesthetic exposure and clinical outcomes at 90-days follow-up in mechanically ventilated patients undergoing EVT for large vessel occlusion in the anterior circulation. The longer patients were exposed to anesthetics, the lower the patients' probability of achieving functional independence and survival was after stroke. Notably, these associations appeared not be driven by peri-procedural anesthesia, suggesting time-dependent mechanisms playing a role in its pathophysiological effects on ischemic brain.

So far, potential detrimental effects of GA on stroke outcomes have been largely linked to its hemodynamic adverse effects such as systemic hypotension and compromise of collateral blood flow to the ischemic brain tissue. The recent randomized SIESTA, ANSTROKE, and GOLIATH trials, applying standardized protocols with specified blood pressure targets and other aspects of hemodynamic management, strongly support these pathophysiological considerations (5–7). However, there is an ongoing discussion whether central nervous system could also be vulnerable to anesthesia-induced neurotoxicity (20, 21). While most of the data originates from animal research, a recent analysis of two observational studies pointed to possible neuronal damage in patients ≥60 years who underwent anesthesia for surgery (22). The analysis revealed a significant increase in both neurofilament light and tau, biomarkers of neuronal damage in the central nervous system, following surgical anesthesia compared to samples taken prior to the procedure. Although these results need be interpreted with caution as it remains unclear whether increases in biomarkers were associated with either anesthesia or surgery, there is still room for speculation of the potential impact of anesthetics on the injured brain in ischemic stroke patients. Perhaps future studies should implement serial analyses of neurofilament light and tau biomarkers in patients undergoing EVT under both GA and CS to further explore their relevance in relation to sedation modality and clinical stroke outcomes.

For the first time, our analysis points toward a time-dependent relationship between anesthetic exposure and unfavorable stroke outcomes in patients undergoing EVT, which is not contradicted by the results of the recent randomized GA vs. sedation trials (5–7). More specifically, the ANSTROKE and GOLIATH trials, which both found no differences between GA and conscious sedation in neurological outcomes 3 months after stroke, achieved an exceptionally high rate (94.5%) of immediate extubation (and therewith cessation of anesthesia) after EVT in GA patients (6, 7). Potential anesthesia-induced adverse effects were therefore limited to the time required for neuro-interventional procedure in the vast majority of patients. When we only considered patients whose anesthetic exposure was limited to peri-procedural time needed, we also found no differences in clinical outcomes between the groups. However, most of our GA patients required continuation of sedation following EVT procedure to keep patients comfortable and safe while intubated that might have influenced functional outcomes unfavorably in our patients. Our observations corroborate a recent analysis on ventilation duration in EVT-treated stroke patients that showed no association between prolonged ventilation and unfavorable functional outcome, as long as ventilation did not exceed 24 h (14). Although specific anesthetic exposure time metrics were not reported in this study, ventilation was likely accompanied by sedation to some extent, supporting the hypothesis of time-varying predictor effects of anesthesia. The SIESTA trial on the contrary reported comparable neurological outcome among GA and CS patients, yet ventilation duration was also prolonged with 49% of GA patients being extubated >2 h after the end of anesthesia (5). However, according to the strict protocol adopted in the SIESTA trial, anesthesia was ended immediately after the EVT or once the patients was admitted to intensive care unit and maintenance of ventilation does not necessarily require continuation of sedation.

Our data is in line with the recently published HERMES individual patient-data meta-analysis and observational studies that showed better clinical outcomes for ischemic stroke patients who underwent EVT under CS or local anesthesia in comparison with those under GA (3, 4, 8). Since neither the meta-analytically considered trials nor the former observational studies specified anesthesia details, it is difficult to conclude whether in these studies proposed anesthesia-induced effects were frontloaded or rather mediated by dose accumulation.

Several limitations of our study need to be acknowledged. First, some patients' necessity of prolonged sedation could have resulted from a medical condition that may have skewed our results, yet we primarily excluded patients who required sedation for more than or equal to 72 h to minimize potential confounding by indication bias. Moreover, baseline clinical and imaging variables appeared well-balanced among anesthesia exposure duration strata and by reviewing indications for prolonged sedation, it appeared that no serious medical condition has driven the corresponding decision on sedation discontinuation in our patient sample. Nonetheless, since our results are entirely based on observational data, confounding may still exist in our data set and our results can be considered only hypothesis generating. Second, hemodynamic monitoring during EVT and afterwards in the intensive care unit followed current stroke guidelines and our institutional protocol (that addressed prevention of both hypoperfusion and hyperperfusion injury, respectively), yet it was not specific as it was in the randomized GA vs. sedation trials (5–7). However, a recent sub-analysis of the SIESTA trial did not show an association between peri-interventional blood pressure drops and early neurological improvement or long-term functional outcome in patients undergoing EVT under GA (23). Although our data does not provide mechanistically insights into anesthetic-induced effects associated with unfavorable outcomes in our analysis, time-dependency of our observation, however, allows the hypothesis that accumulating direct or indirect action of anesthetics on the brain might play a certain predictive role. Third, our data cannot be used to restrict observed associations to either inhalational or intravenous anesthetics as both were utilized in our patients. Also, from our data we cannot draw any conclusions on effects if patients were treated without sedation at all (e.g., local anesthesia only). Our study also does not allow to differentiate between effects of anesthetics and those of mechanical ventilation on patients' outcomes. Lastly, the study's monocentric nature could limit external validity of our findings. On the other hand, our study also has certain strengths including the utilization of registry data, the relatively large sample of consecutive patients undergoing EVT under GA in a real-world scenario and the first-ever analysis of length of anesthetic exposure and its potential impact on stroke outcomes.

In conclusion, in ischemic stroke patients undergoing EVT for large vessel occlusion, patients who require peri-interventional GA should be extubated as soon as possible after the procedure. Further research is needed to determine whether continued sedation needed post-EVT rather than peri-interventional GA *per se* is associated with unfavorable clinical outcomes in these patients.

## Data Availability

The datasets for this study will not be made publicly available because consent statements of study patients and local data protection guidelines restrict its use to investigators of the Technische Universität Dresden, Germany, and their affiliates.

## Ethics Statement

The study complied with The Strengthening the Reporting of Observational Studies in Epidemiology (STROBE) statement and has been approved by the institutional research ethics committee of the Technische Universität Dresden (#272072017). Since we used observational data from an ongoing registry informed formal consent was not required; however, patients or their legally authorized representatives gave approval for treatment with intravenous thrombolysis and/or endovascular therapy, where possible.

## Author Contributions

LR: project development, data collection, and manuscript writing. HM: data collection and manuscript editing. AP, JG, HT, TS, LP, and SW: data collection and manuscript revising. JB: project development, data collection, and manuscript writing/revising. KH: project development and data collection. HR: project development and manuscript revising. JL: project development, data collection and manuscript revising. VP and KB: project development, data collection, manuscript writing/revising and data analysis.

### Conflict of Interest Statement

The authors declare that the research was conducted in the absence of any commercial or financial relationships that could be construed as a potential conflict of interest.
